# Astragaloside IV inhibits lung cancer progression and metastasis by modulating macrophage polarization through AMPK signaling

**DOI:** 10.1186/s13046-018-0878-0

**Published:** 2018-08-29

**Authors:** Fei Xu, Wen-Qiang Cui, Ying Wei, Jie Cui, Jian Qiu, Ling-Li Hu, Wei-Yi Gong, Jing-Cheng Dong, Bao-Jun Liu

**Affiliations:** 1 Department of Integrative Medicine, Huashan Hospital, Fudan University, Shanghai, China; 2 Institutes of Integrative Medicine, Fudan University, Shanghai, China; 3 Department of Integrative Medicine and Neurobiology, State Key Laboratory of Medical Neurobiology, Institute of Acupuncture Research, School of Basic Medical Science, Fudan University, Shanghai, China

**Keywords:** Tumor-associated macrophages, Macrophage polarization, Astragaloside IV, Lung cancer

## Abstract

**Background:**

Accumulating evidence suggests that M2-polarized tumor-associated macrophages (TAMs) play an important role in cancer progression and metastasis, making M2 polarization of TAMs an ever more appealing target for therapeutic intervention. Astragaloside IV (AS-IV), a saponin component isolated from *Astragali radix*, has been reported to inhibit the invasion and metastasis of lung cancer, but its effects on TAMs during lung cancer progression have not been investigated.

**Methods:**

Human THP-1 monocytes were induced to differentiate into M2 macrophages through treatments with IL-4, IL-13, and phorbol myristate acetate (PMA). We used the lung cancer cell lines A549 and H1299 cultured in conditioned medium from M2 macrophages (M2-CM) to investigate the effects of AS-IV on tumor growth, invasion, migration, and angiogenesis of lung cancer cells. Macrophage subset distribution, M1 and M2 macrophage-associated markers, and mRNA expression were analyzed by flow cytometry and quantitative PCR. The activation of adenosine monophosphate-activated protein kinase (AMPK) signaling pathways that mediate M2-CM–promoted tumor migration was detected using western blotting.

**Results:**

Here we found that AS-IV significantly inhibited IL-13 and IL-4–induced M2 polarization of macrophages, as illustrated by reduced expression of CD206 and M2-associated genes, and that AS-IV suppressed the M2-CM–induced invasion, migration, and angiogenesis of A549 and H1299 cells. In vivo experiments demonstrated that AS-IV greatly inhibited tumor growth and reduced the number of metastases of Lewis lung cancer. The percentage of M2 macrophages was decreased in tumor tissue after AS-IV treatment. Furthermore, AS-IV inhibited AMPKα activation in M2 macrophages, and silencing of AMPKα partially abrogated the inhibitory effect of AS-IV.

**Conclusions:**

AS-IV reduced the growth, invasion, migration, and angiogenesis of lung cancer by blocking the M2 polarization of macrophages partially through the AMPK signaling pathway, which appears to play an important role in AS-IV’s ability to inhibit the metastasis of lung cancer.

**Electronic supplementary material:**

The online version of this article (10.1186/s13046-018-0878-0) contains supplementary material, which is available to authorized users.

## Background

Lung cancer is the leading cause of cancer-related deaths and is the most common cancer among both men and women worldwide [1] in spite of recent advances in lung cancer diagnosis and treatment, especially targeted therapy for advanced-stage lung cancer guided by next-generation sequencing [2]. The five-year survival rate has failed to improve significantly over the last 30 years and remains a mere 18%, which is much lower than that of other common cancers [3, 4], due to recurrence and metastasis, thus emphasizing the need to better understand the molecular mechanisms of lung cancer in order to suggest novel therapeutic targets.

Collective evidence demonstrates that the tumor microenvironment (TME) plays an important role in lung cancer pathogenesis [5, 6]. M2-polarized macrophages are commonly called tumor-associated macrophages (TAMs), and these promote cancer cell growth, invasion, metastasis, and angiogenesis and are one of the primary tumor-infiltrating immune cells. Several lines of evidence suggest that TAMs not only open up avenues for the initiation and metastasis of tumor cells, but they also exhibit immunosuppressive activities and promote tumor angiogenesis [7]. Recent large longitudinal studies have reported that TAM infiltration in the tumor site frequently correlates with poor prognosis of various cancers [8, 9]**.** Thus, M2-polarized macrophages are considered to be a potential target for adjuvant anticancer therapies, and recent therapeutic approaches targeting the M2 polarization of TAMs have shown encouraging results. For example, the tumor burden of tumor-bearing mice treated with clodronate-encapsulated liposomes decreased by 50% compared with vehicle-treated mice, and tumor cell proliferation was also attenuated [10].

Traditional Chinese medicine (TCM) has gained increasing acceptance worldwide with its advantages of low toxicity, limited side effects, and good tolerance [11]. Of note, there is evidence that TCM has an indispensable role in cancer prevention and treatment by preventing tumorigenesis, by attenuating the toxicity and enhancing the effect of treatments such as radio- and chemotherapy, and by reducing tumor recurrence and metastasis [12]. Importantly, it has been irrefutably shown that TCM is a potential treatment strategy for regulating the TME [13]. Astragaloside IV (AS-IV, 3-O-β-D-xylopyranosyl-6-O-β-D-glucopyranosyl cycloastragenol), a natural saponin extracted from *Astragali radix*, has been reported to have anti-inflammatory, anti-cancer, anti-oxidative, and immune-regulatory effects [14–16]*.* However, if and how AS-IV exerts anti-tumor effects through modulating the inflammatory microenvironment or acting on immune cells has not been reported. In the present study, we investigated the relationship between macrophage polarization and the antitumor effect of AS-IV in vitro and in vivo. Here we show that AS-IV acts directly on macrophages to inhibit macrophage polarization to the M2 phenotype and that it suppresses the invasion, migration, and angiogenesis of lung cancer cells by modulating the AMPK signaling pathway.

## Methods

### Reagents

AS-IV powder (C_41_H_68_O_14_, molecular weight: 784.9702, purity > 98%) was purchased from Shanghai Ronghe Co. (Shanghai, China). The following antibodies were purchased from eBiosciences (CA, USA): anti-human CD206 PE, anti-human CD86 PE, anti-human CD14 PerCP/Cy5.5, anti-mouse CD45 v450, anti-mouse CD11b FITC, anti-mouse F4/80 PE, anti-mouse CD206 Alexa-647, and anti-mouse iNOS APC. Antibodies against total and phosphorylated AMPKα, arginase 1 (Arg-1), CD31, and vascular endothelial growth factor A (VEGFA) were purchased from Cell Signaling Technology (MA, USA). IL4, IL13, IFN-γ, and LPS were purchased from Peprotech (NJ, USA). Phorbol 12-myristate 13-acetate (PMA) was purchased from Liankebio (Hangzhou, China). JetPrime transfection agent was obtained from Polyplus (NY, USA).

### Cell lines and cell culture

The human lung cancer cell lines A549 and H1299, the human monocyte cell line THP-1, and Lewis lung cancer (LLC) cells were purchased from the Cell Bank of the China Science Academy (Shanghai, China). A549 cells and LLC cells were cultured in DMEM and H1299 and THP-1 cells were maintained in RPMI-1640 medium (Gibco, Grand Island, NY, USA), and all cultures were supplemented with 10% fetal bovine serum (Gibco) and 100 U per ml of penicillin-streptomycin (KeyGEN BioTECH, Nanjing, China, KGY002–50) and kept under 5% CO_2_ at 37 °C.

### Macrophage polarization

The THP-1 cells were differentiated into M0 macrophages by incubating in 320 nmol/L PMA for 18 h. To obtain M1-polarized macrophages, THP-1 cells were treated with 320 nmol/L PMA for 12 h and then cultured with 100 nmol/L PMA plus 100 ng/mL lipopolysaccharide (LPS) and 20 ng/mL IFN-γ for a further 48 h. To generate M2-polarized macrophages, THP-1 cells were treated with 320 nmol/L PMA for 12 h and then cultured with 100 nmol/L PMA plus 20 ng/mL IL-4 and 20 ng/mL IL-13 for a further 48 h.

### Cell treatment

AS-IV was dissolved in dimethyl sulfoxide (DMSO) for the treatment of macrophages. The final concentration of DMSO was less than 0.1% (*v*/v). For treatment with AS-IV, cells were incubated for 48 h with or without IL-4/IL-13. To exclude the effects of DMSO, the adherent macrophages were treated with DMSO (0.1%) alone for 48 h.

### MTT assays

After exposure to various concentrations of AS-IV, the viability of cells was determined using the MTT assay system (Beyotime Institute of Biotechnology, Shanghai, China). In brief, the cells were plated on 96-well culture plates at a density of 1 × 10^6^ cell/ml. After the specific timeframe, we added 10 μL MTT reagent to each well and then cultured the cells for 4 h. Afterwards, 100 μL formazan solution was added to each well. The 96-well culture plate was agitated for 10 min on a shaker. Finally, the OD value of each well was detected at 490 nm using a MK3ELISA Reader (Thermo fisher scientific, USA). Cell viability was calculated as follows: treated group OD/control group OD × 100%.

### Flow cytometry

Mononuclear cells were isolated from tumor tissue from animal model by cutting the tissue into pieces; digesting the pieces with DNAses, collagenase II, and collagenase IV; pushing them twice through 300 mesh screens; and then treating them with erythrocytolysin. Cells were then stained with antibodies against CD45, CD11b, F4/80, CD206, and iNOS for 30 min at 4 °C in the dark, then washed twice and resuspended in 500 μL of phosphate-buffered saline (PBS). The cells were sorted on a FACSCalibur cytometer (BD Biosciences,USA) or an Attune NxT (Life Technologies,USA) and analyzed using the FlowJo software (Ashland, OR).

### Cell transfection

SiRNA was produced by Genomeditech, Co. (Shanghai, China) and was used for AMPK gene knockdown. The sequences of siRNAs were as follows: Si-AMPK 1 (SiRNA 1): 5’–ACC CAU AUU AUU UGC GUG U–3′ (forward) and 5’–ACA CGC AAA UAA UAU GGG U–3′ (reverse); Si-AMPK 2 (SiRNA 2): 5′ –CAC AGC CAA AUG CUU CCA U–3′ (forward) and 5’–AUG GAA GCA UUU GGC UGU G–3′ (reverse); Si-AMPK 3 (SiRNA 3): 5’–UAG AAG UCA AAG UCG ACC A–3′ (forward) and 5’–UGG UCG ACU UUG ACU UCU A–3′ (reverse). A scrambled siRNA was used as the negative control. About 5 × 10^5^ cells per well were seeded in 6 well plates, and after PMA stimulation the non-adherent cells were then washed away. Transfection of siRNA into the macrophages was performed using jetPrime (Polyplus) according to the manufacturer’s recommendations. Cells were transfected with 40 nmol/L siRNA for 12 h, 24 h, and 48 h, and then siRNA transfection in the best condition was performed. The knockdown efficiency of Si-AMPK was tested by quantitative PCR assay and western blot analysis.

### Real-time PCR assay (RT-PCR)

Total RNA from cells was isolated using Trizol (Sigma), and cDNA was synthesized using the Prime Script RT reagent Kit (Takara). The sequences of the primers used for the RT-PCR are listed in Table 1. The quantitative real-time RT-PCR was carried out in a 6-well plate using the Takara SYBR Green reagents on an Applied Biosystems 7300 plus according to the manufacturer’s protocol.

**Table 1:** Hematological parameters in blood

	NS	AS-IV	*p* value
WBC (× 10^9^/L)	10.75 ± 0.76	10.58 ± 0.89	0.89
neutrophils (%)	45.50 ± 3.11	45.73 ± 2.54	0.96
monocytes (%)	4.17 ± 0.23	4.40 ± 0.35	0.61
Leukocytes (%)	50.33 ± 3.31	59.87 ± 8.52	0.36
RBC (%)	9.12 ± 0.75	9.55 ± 0.71	0.70
NLR	91.92 ± 11.53	78.62 ± 8.97	0.41
PLR	8.40 ± 0.95	8.12 ± 1.20	0.86
MCH (pg)	13.43 ± 0.18	12.97 ± 1.53	0.78
MCV (fl)	51.89 ± 0.57	53.49 ± 1.80	0.43
Hgb (g/L)	123.00 ± 8.74	113.00 ± 14.36	0.58

All of the data are presented as the mean ± SEM. N = 6~ 7 animals per group

Abbreviation: *WBC* white blood cell, *RBC* red blood cell, *NLR* neutrophil-lymphocyte ratio, *PLR* platelet-lymphocyte ratio, *MCH* mean corpuscular hemoglobin, *MCV* mean corpuscular volume, *Hgb* hemoglobin, *SEM* standard error of mean

### Conditioned medium preparation

Different polarized macrophages were incubated in serum-free medium for 24 h and then centrifuged at 10,000 rpm for 5 min, after which supernatants were collected as conditioned medium and stored at − 80 °C.

### Wound healing assay

Cells were cultured on 6-well plates (4 × 10^5^ cells/well), and when adhering to the wall a monolayer culture with a space without cells was obtained by scratching horizontally across the wall with a disposable pipette tip. Dislodged cells were washed away with PBS three times and aspirated. The cells were incubated in serum free medium or M2-CM and with or without AS-IV. After incubation for 48 h, cell invasion was observed and photographed using a phase contrast inverted microscope. Three random fields along the scraped line were selected and analyzed with ImageJ software.

### Invasion assay

The invasion assay was performed in a 24-well cell culture chamber using inserts with 8 μm pores (Corning). Inserts containing 2 × 10^5^ A549 or H1299 cells were transferred to wells containing 5 × 10^5^ M0 macrophages, M2 macrophages, or M0 and M2 macrophages and cultured with AS-IV for 48 h. After incubation, cells on the upper surface were removed. Cells on the reverse side were fixed with 4% paraformaldehyde for 15 min and then stained with crystal violet. Finally, the invasive cells were counted under a microscope at 200× magnification.

### Cytokine analysis

IL-10 and TGF-β levels in M0 and M2 macrophages with and without AS-IV were measured using enzyme-linked immunosorbent assay (ELISA) kits (RayBiotech) according to the manufacturer’s instructions.

### Western blot analysis

Different macrophages in 6-well plates (about 5 × 10^5^ cells/well) were harvested in lysis buffer and incubated for 30 min at 4 °C. Supernatants were obtained after being centrifuged at 12,000 rpm for 20 min and then quickly frozen. The protein concentration was measured by bicinchoninic acid assay (Thermo Scientific). About 30 μg of protein was electroblotted onto a PVDF membrane following electrophoretic separation on a 10% SDS-polyacrylamide gel. The immunoblot was incubated for 2 h with 5% non-fat milk at room temperature and subsequently incubated overnight at 4 °C with a 1:1000 dilution of AMPKα and p-AMPKα antibodies and 1:10,000 dilution of GAPDH antibody. Blots were washed three for 15 min with Tris-Buffered Saline and 0.1% Tween 20 (TBST) and then incubated with a 1:1000 dilution of HRP-conjugated secondary antibody for 2 h at room temperature. Blots were again washed three times for 15 min with TBST and then developed by enhanced chemiluminescence (Thermo Scientific).

### Animal model

Male C57BL/6 J mice (5 weeks old) were purchased from Shanghai SLAC Co. (Shanghai, China) and were randomly divided into two groups. In the subcutaneous model, 2 × 10^6^ LLC cells (resuspended in cold PBS to a final concentration of 1 × 10^7^ cells/ml) in 0.2 ml PBS were injected subcutaneously into the right side of the back of each mouse. After 10 days, tumor size was measured twice weekly by a digital caliper and was calculated as (D^2^ × d) / 2, where D is the large diameter and d is the small diameter of the tumor. In the intravenous model, 2 × 10^6^ LLC cells in 0.2 ml PBS were injected into the tail vein of each mouse. In both models, after one day the mice were given an intragastric (i.g.) administration of 0.3 mL AS-IV (40 mg/kg) for 21 consecutive days. Control animals received equal volumes of normal saline (NS). On day 22 the animals were sacrificed and the tumors were removed and immediately weighed. However, to observe the overall survival of mice, AS-IV and NS was respectively continued to be given throughout the life of the animal until they died.

### Immunohistochemistry (IHC)

IHC staining was used to investigate the macrophage changes in the tumor tissues. Endogenous peroxidase blockage and heat-induced epitope retrieval were performed according to the manufacturer’s protocol. The expression of F4/80, CD206, CD31, and VEGFA was quantitatively evaluated using an Olympus Cx31 microscope with the Image-Pro Plus medical image analysis system. The positive cells were evaluated by ImageJ software, and all measurements were performed in three randomly selected microscopic fields for each slide.

### Fluorescence confocal microscopy

A total of 5 × 10^5^ cells were cultured in 6-well plates and were fixed with 4% paraformaldehyde for 30 min at room temperature (RT) and washed three times with 5% PBS-Tween (PBST) for 15 min. After washing, the cells were blocked in 1% BSA at RT and then stained with anti-STAT3 antibody (1:50 dilution) in 1% BSA at 4 °C overnight. The cells were then washed three times with 5% PBST for 15 min and stained with an Alexa Fluor-488 secondary antibody (1:1000, dilution; Abcam, Cambridge, UK) for 2 h at RT. The cells were again washed three times with 5% PBST for 15 min. After desiccation, the cells were counterstained with DAPI (Sigma, St. Louis, MO).

Tumor tissues were removed and fixed in 4% paraformaldehyde at 4 °C for 3 days and then dehydrated in 30% sucrose and finally embedded in paraffin. The tissues were then cut into 5 μm-thick serial sections and incubated with anti-Rabbit CD31 (5 μg/ml, Arigo, 52,748, Taiwan, China) or anti-VEGFA (5 μg/ml, Arigo, 10,513, Taiwan, China) antibodies and then stained with an Alexa Fluor-549 or − 488 conjugate secondary antibody (1:1000 dilution). All cells were visualized with a laser confocal scanning microscope.

### Statistical analysis

Data were expressed as means ± standard error of mean (SEM). The statistical significance of the differences was tested using one-way analysis of variance (ANOVA) or Student’s t-tests. A *p*-value less than 0.05 was regarded as statistically significant. All statistical analyses were performed in SPSS v19.0.

## Results

### Establishment of human M1 and M2-polarized macrophages

**Fig. 1:**
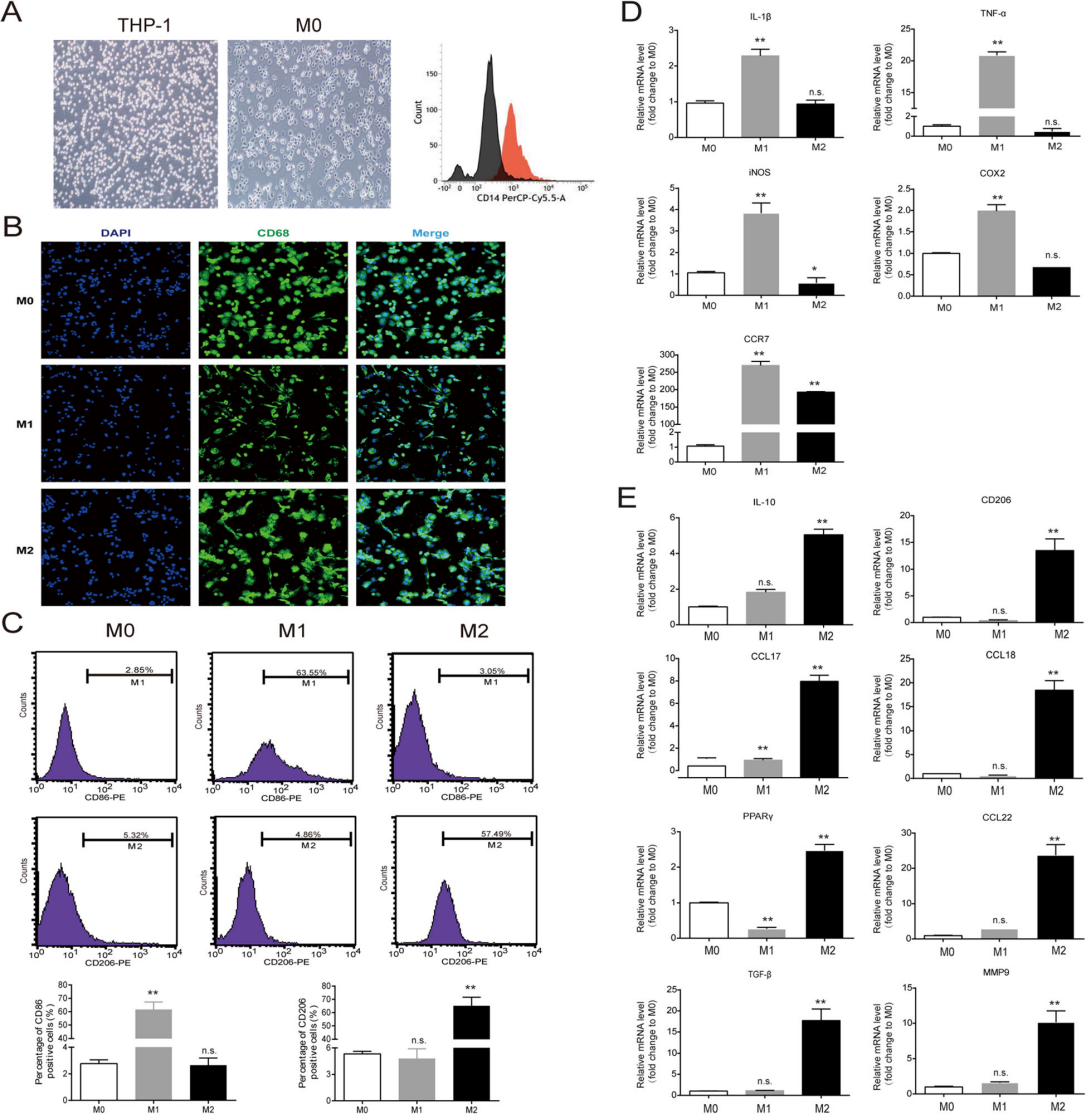
Human THP-1 monocyte differentiation into M1 and M2 macrophages. **a** The morphology of M0 (non-polarized THP-1 cells) and the expression of CD14 as measured by flow cytometry. **b** Confocal microscopy showing the distinct morphology of THP-1–derived M1 and M2 macrophages stained with DAPI (blue) and with antibodies against CD68 (green). **c** Flow cytometry demonstrated cell surface expression of CD86 and CD206 in the M0, M1, and M2 populations. **d** and **e** The mRNA expression levels of M1 and M2 macrophage markers were measured by QT-PCR. Data are presented as the mean ± SEM from three independent experiments. All results show the relative fold change compared to M0 (non-polarized THP-1 cells). *p < 0.05, **p < 0.01, and n.s. = no significance. IL, interleukin; TNF-α, tumor necrosis factor-α; iNOS, inducible nitric oxide synthase; CCR, chemokine receptor; CCL, C-C motif chemokine; MMP, matrix metalloproteinase

To generate a model of macrophage polarization, we used PMA-differentiated human THP-1 monocytes (M0 macrophages) stimulated by LPS and IFN-γ (M1 polarized macrophages) or IL-4 and IL-13 (M2 polarized macrophages) as representative of the two opposite polarized states. As shown in Fig. 1a and b, THP-1 monocytes incubated in the presence of PMA became adherent and rounded, and the expression of the recognized macrophage markers CD14 and CD68, as analyzed by flow cytometry and immunofluorescence staining, respectively, confirmed the monocyte-to-macrophage differentiation that was characterized by decreased CD14 expression and increased CD68 expression. M1 macrophages showed cellular elongation in cell morphology, while M2 macrophages showed a rounded morphology. The surface markers CD86 (M1 phenotype) and CD206 (M2 phenotype) were analyzed by flow cytometry, and increased expression of CD86 and CD206 was seen in the M1 macrophages and the M2 macrophages, respectively (Fig. 1c). As expected, the mRNA expression of *IL-1β*, *TNF-α*, *iNOS*, *COX2*, and *CCR7* (M1 macrophage markers) was significantly upregulated in the M1 population (Fig. 1d), and the mRNA expression of *IL-10*, *CD206*, *CCL17*, *CCL18*, *PPARγ*, *CCL22*, *TGF-β*, and *MMP9* (M2 macrophage markers) was greater in the M2 population (Fig. 1e). Taken together, these findings indicated the successful polarization of monocytes into M1 and M2-polarized macrophages.

### AS-IV suppressed M2 polarization of macrophages in vitro

**Fig. 2:**
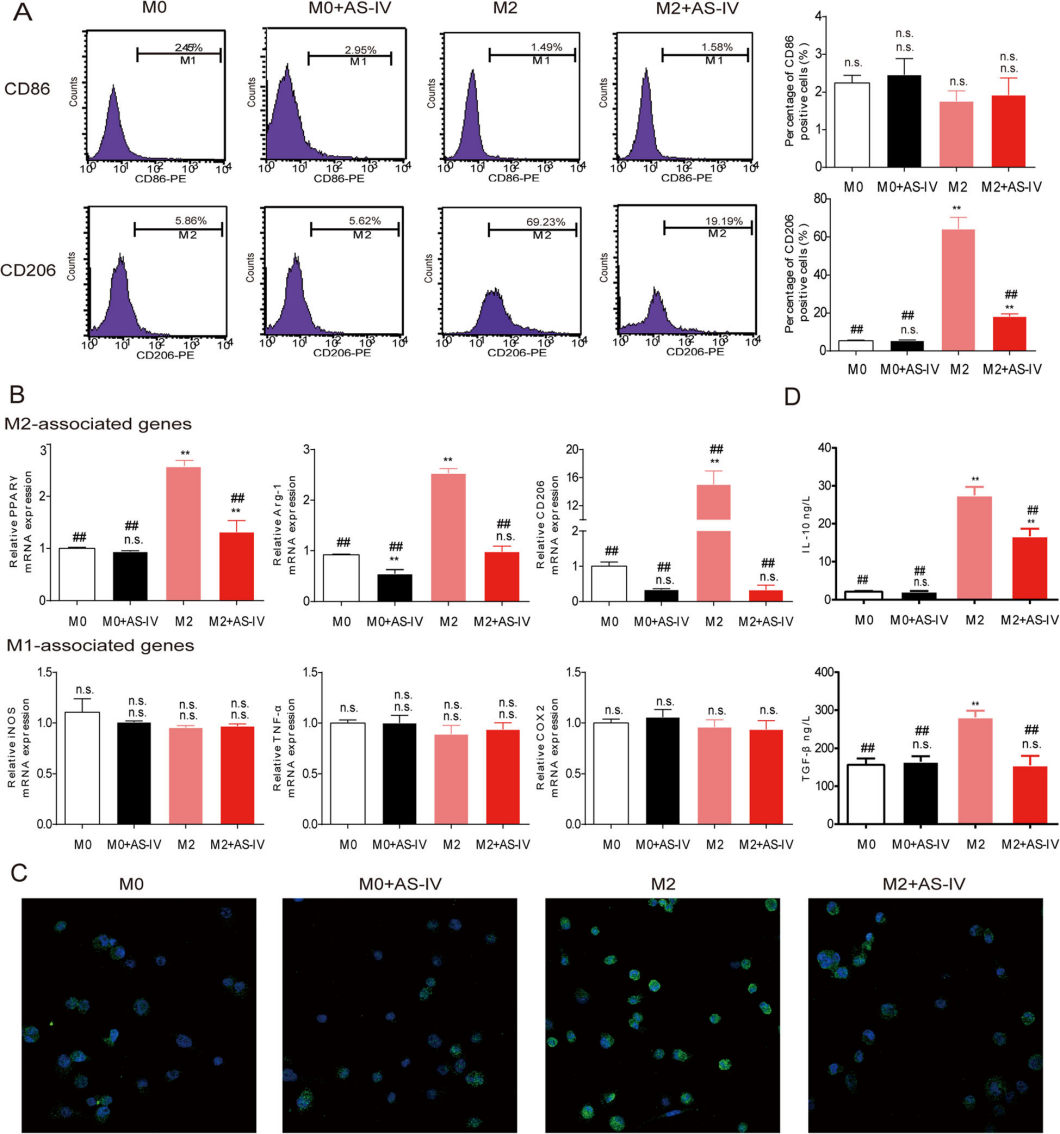
AS-IV inhibited macrophage M2 polarization. Macrophages derived from THP-1 cells were stimulated with IL-4/IL-13 with or without AS-IV (80 nM) for 48 h. **a** Flow cytometry was used to quantify the expression of CD206, an M2 macrophage marker, and CD86, an M1 marker. **b** QT-PCR was performed to detect gene levels in M2 and M1 macrophages. M2-associated genes included *PPARγ*, *Arg-1*, and *CD206*, and M1-associated genes included *iNOS*, *TNF-α*, and *COX2*. **c** Representative images of M0 and M2 macrophages with and without AS-IV stained with antibodies against Arg-1 (green) and with DAPI (blue). **d** The levels of IL-10 and TGF-β in the cell culture supernatants were measured by ELISA. Data are presented as the mean ± SEM from three independent experiments. Compared to M0, **p < 0.01, *p < 0.05, and n.s., no significance; compared to M2, ##*p* < 0.01, #*p* < 0.05, and n.s., no significance

To determine whether AS-IV inhibits IL-4 and IL-13–induced macrophage M2 polarization, we first measured the expression of the surface markers CD86 and CD206 by flow cytometry in M0 and M2 macrophages following treatment with AS-IV. As shown in Fig. 2a, significant upregulation of CD206 was observed when THP-1 monocytes were treated with IL-4 and IL-13 for 72 h, and this was greatly reduced by AS-IV in a concentration-dependent manner (Additional file 1: Figure S1A). Meanwhile, the cytotoxicity of AS-IV to macrophages was evaluated by the MTT assay, which showed that AS-IV did not cause significant cell death at concentrations from 40 μM to 100 μM (Additional file 1: Figure S1B). To further confirm the effect of AS-IV on M2 polarization, the mRNA level of surface markers for macrophages was assessed by real-time PCR. The expression of M2 macrophage markers, including *PPARγ*, *Arg-1*, and *CD206* were decreased by 80 μM AS-IV, while the M1 macrophage markers, including *TNF-α*, *iNOS*, and *COX-2* were not affected (Fig. 2b). DMSO alone had no effect on M2 polarization (Additional file 2: Figure S2A and B). Confocal microscopy showed that treatment with AS-IV decreased the expression of the Arg-1 protein (Fig. 2c), and AS-IV significantly reduced the levels of IL-10 and TGF-β in the supernatants from M2 macrophage cultures (Fig. 2d). Taken together, these results suggest that AS-IV effectively inhibits M2 macrophage polarization in vitro*.*

### AS-IV inhibited the migration and invasion of tumor cells and inhibited the angiogenesis- promoting phenotype of M2 macrophages

**Fig. 3:**
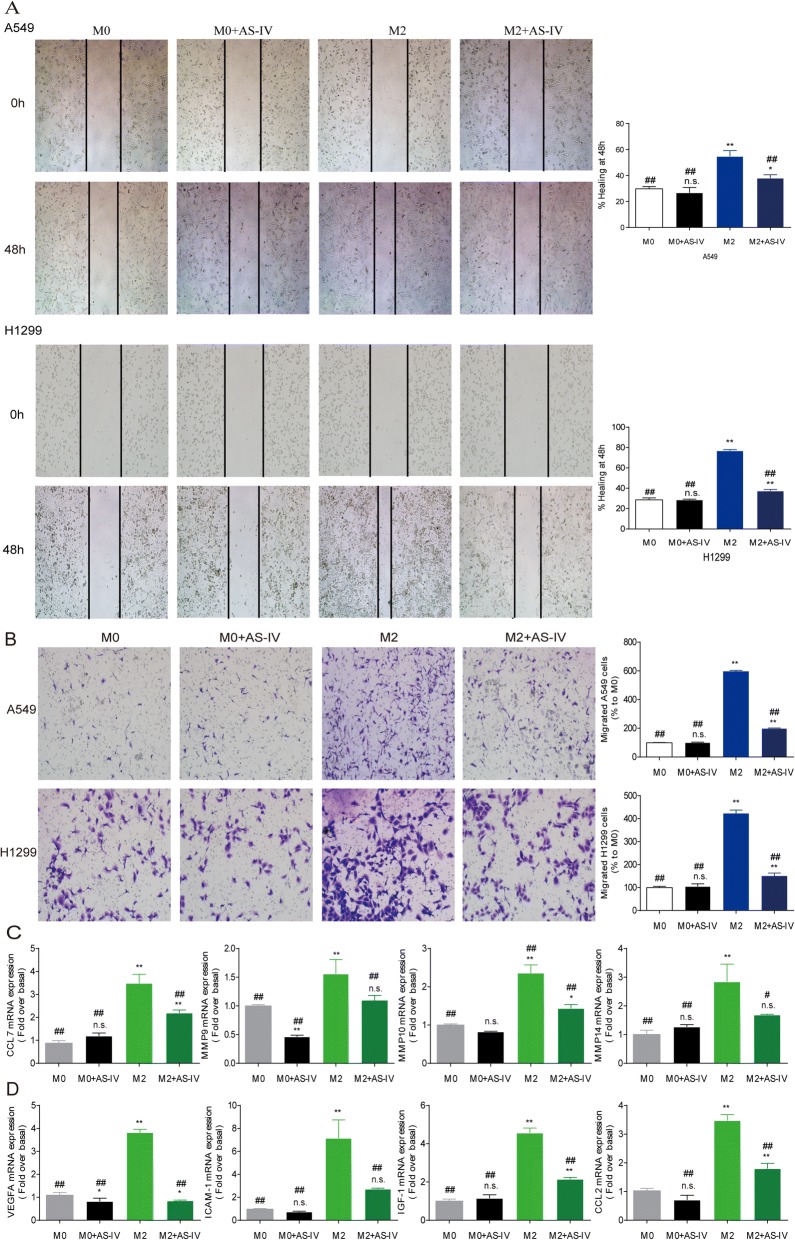
AS-IV inhibited the migration and invasion of tumor cells. Macrophages derived from THP-1 were stimulated with IL-4/IL-13 with or without AS-IV (80 nM) for 48 h, and the conditioned medium (CM) was collected. A549 and H1299 cells were cultured with different CM. (**a** and **b**) The effect of AS-IV on A549 and H1299 invasion and migration was evaluated by wound scratch assay and trans-well assay, respectively. (**c** and **d**) The mRNA levels of genes associated with migration and angiogenesis in macrophages. Data are presented as the mean ± SEM from three independent experiments. Compared to M0, **p < 0.01, *p < 0.05, and n.s., no significance; compared to M2, ^##^*p* < 0.01, ^#^*p* < 0.05, and n.s., no significance

There is a growing body of evidence that M2-polarized macrophages can promote tumor migration, invasion, metastasis, and angiogenesis and can activate tumor-promoting genes in cancer cells [10, 17], therefore we sought to determine whether AS-IV affects lung cancer cells treated with M2-CM. M2-CM significantly promoted the migration of A549 and H1299 cells in wound-healing assays, whereas M2-CM from combined treatment with AS-IV significantly reduced this effect (Fig. 3a). We also observed that M2-CM significantly increased cell invasion in A549 and H1299 cells in comparison with M0-CM, and this effect on cell migration was also abrogated by AS-IV (Fig. 3b). Furthermore, the expression of metastasis-related genes, including *CCL7*, *MMP9*, *MMP10*, and *MMP14*, was significantly increased in A549 and H1299 cells treated with M2-CM (Fig. 3c), and AS-IV partially blocked this increase. In addition, M2-CM treatment induced mRNA expression of angiogenesis-promoted genes, including *VEGFA*, *ICAM-1*, *IGF-1*, and *CCL2*, which was dramatically attenuated by AS-IV (*p* < 0.01, Fig. 3d). Taken together, these results suggest that AS-IV inhibits the M2-CM–induced migration and invasion of lung cancer cells and decreases the angiogenesis-promoting phenotype of M2 macrophages in vitro.

### AS-IV inhibited AMPK signaling pathways in macrophages

**Fig. 4:**
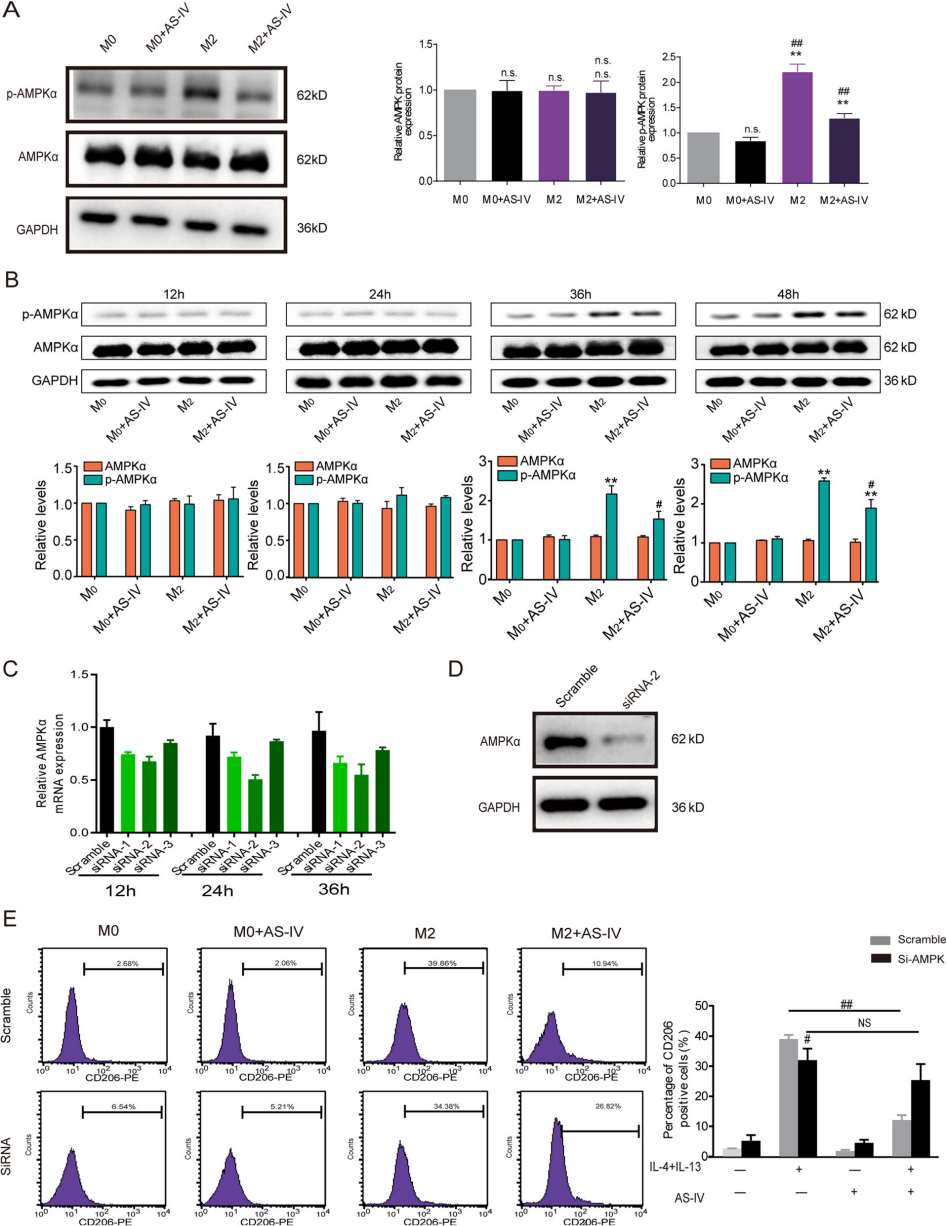
AS-IV treatment inhibited AMPK signaling in the M2 polarization of macrophages. **a** Macrophages derived from THP-1 cells were stimulated with IL-4/IL-13 with or without AS-IV (80 nM) for 48 h. The levels of AMPKα and p-AMPKα as measured by western blot. **b** Macrophages derived from THP-1 cells were stimulated with IL-4/IL-13 with or without AS-IV (80 nM) for 12 h, 24 h, 36 h, and 48 h. AMPKα and p-AMPKα1 levels were measured by western blot. **c** The mRNA levels of AMPKα in macrophages were knocked down by siRNAs. **d** Macrophages were transiently transfected with siRNA 2 for 24 h, and AMPKα protein expression in transfected cells was analyzed by western blot. **e** siRNA2 blocked AMPKα expression. The percentage of CD206-positive macrophages was determined by flow cytometry. All experiments were repeated three times. Compared to M0, **p < 0.01, *p < 0.05, and n.s., no significance; compared to M2, ^##^*p* < 0.01, ^#^*p* < 0.05, and n.s., no significance

We investigated the molecular mechanisms by which AS-IV inhibited macrophage M2 polarization. Western blot analysis indicated that p-AMPKα was up-regulated by IL-4/IL-13 stimulation in macrophages, and this effect was reduced by AS-IV (*p* < 0.01, Fig. 4a). The expression of AMPKα showed no changes upon IL-4/IL-13 treatment, while p-AMPKα was elevated in a time-dependent manner (Fig. 4b). AS-IV reduced this increase, especially at the 48 h time point (Fig. 4b). As shown in Fig. 4c, siRNA was used to knock down AMPKα in macrophages, and siRNA 2 for 24 h had the best effects and was used in subsequent experiments. Western blot analysis (Fig. 4d) showed that transfection with siRNA 2 resulted in a significant decrease in AMPKα levels after 24 h compared with the negative control transfection. When AMPKα expression was suppressed by siRNA 2, the flow cytometry assay showed that AS-IV treatment could no longer reduce M2 macrophage activation (*p* > 0.05, Fig. 4e). These results suggest that the AMPK signaling pathway is involved in the inhibitory effect of AS-IV on M2 polarization of macrophages.

### AS-IV was not cytotoxic to lung cancer cells

**Fig. 5:**
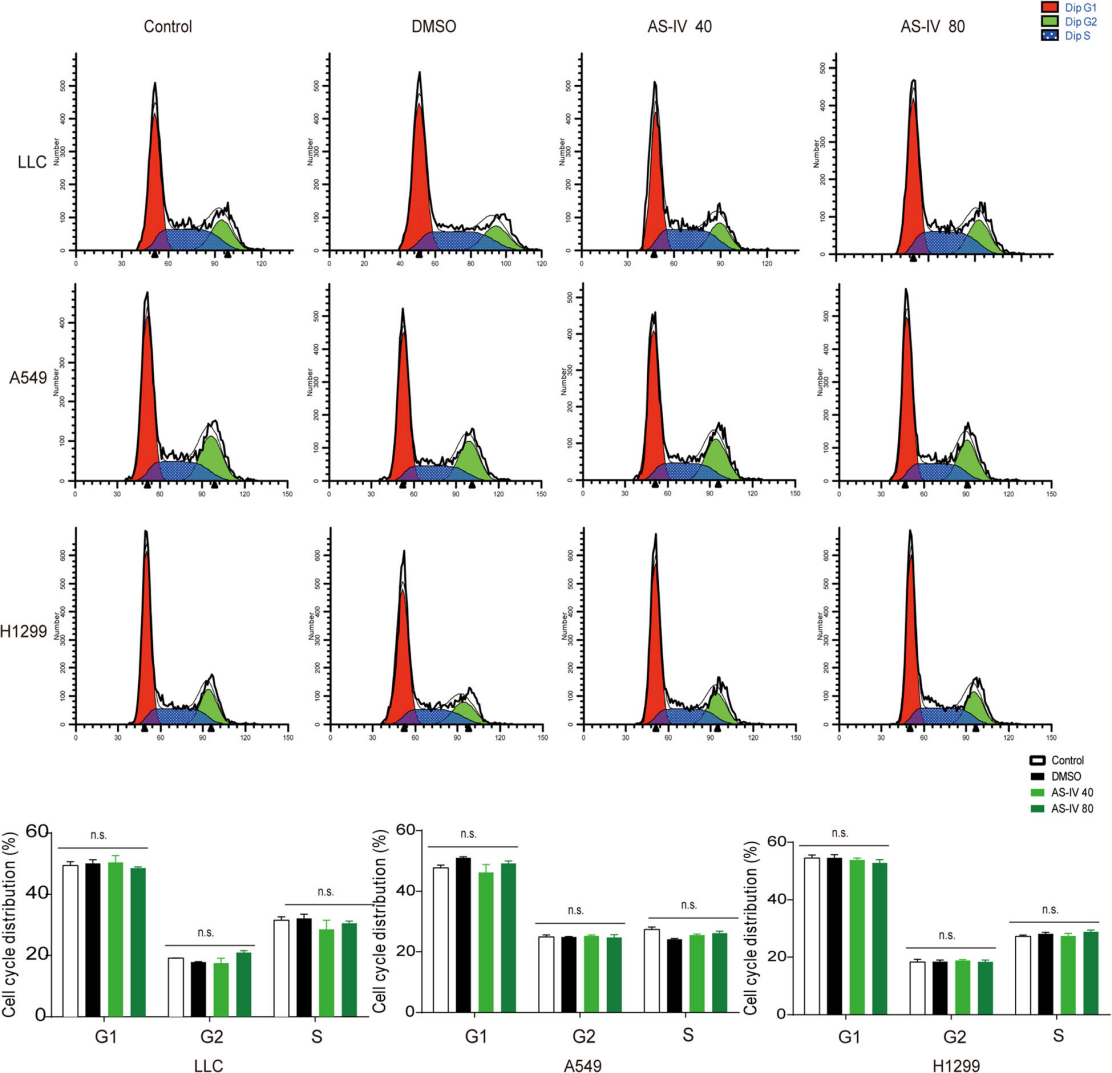
AS-IV was not toxic to tumor cells. LLC, A549, and H1299 cells were treated with blank control, DMSO, 40 μM AS-IV, or 80 μM AS-IV for 48 h. The populations of cells in each phase of the cell cycle were detected by flow cytometry using PI staining. All of the data are presented as the mean ± SEM from three independent experiments

As shown above, AS-IV displayed antitumor effects by regulating M2 polarization, but it was not clear if this effect was because AS-IV is cytotoxic to tumor cells. Flow cytometry showed no significant difference in cell cycle progression of tumor cells among the four groups treated with blank control, DMSO, 40 μM AS-IV, or 80 μM AS-IV (Fig. 5), indicating that the inhibitory effects of AS-IV on migration and invasion of lung cancer cells is unlikely to be due to direct tumoricidal activity.

### AS-IV inhibited tumor growth and metastasis and prolonged survival in tumor-bearing mice in vivo by targeting macrophages

**Fig. 6:**
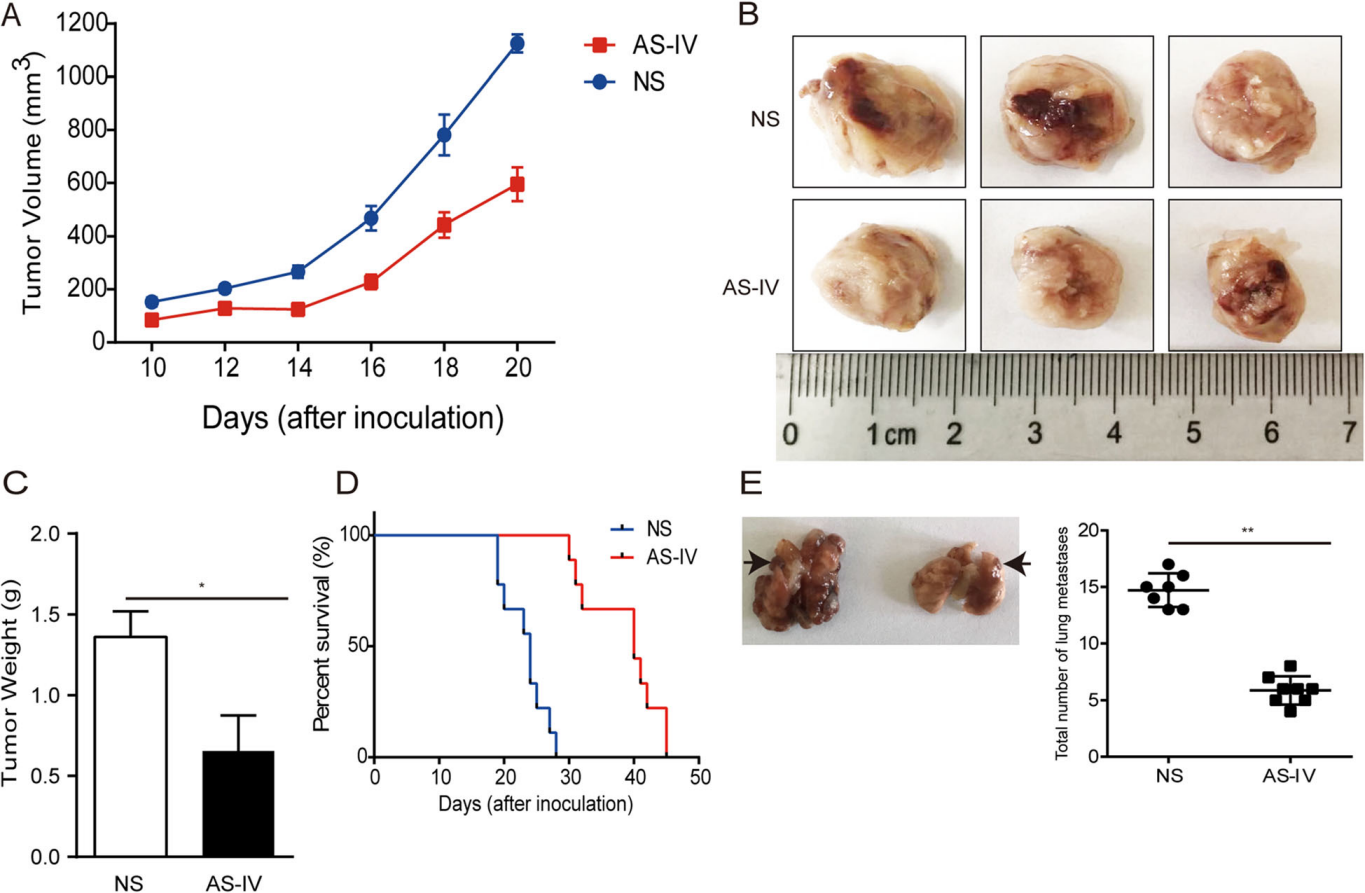
AS-IV inhibited the growth and migration of LLC tumors and prolonged survival *in vivo*. **a** The tumor volumes were determined at different time points. N = 6–15 animals per group. **b** Representative image of tumors at day 22. **c** The weights of isolated tumor tissues were measured. **d** Survival curves. **e** Representative image of lung metastases at day 22 and total number of lung metastases in the intravenous model. Data are presented as the mean ± SEM. *N* = 6–8 animals per group. Compared to the control group, **p < 0.01, *p < 0.05

**Fig. 7:**
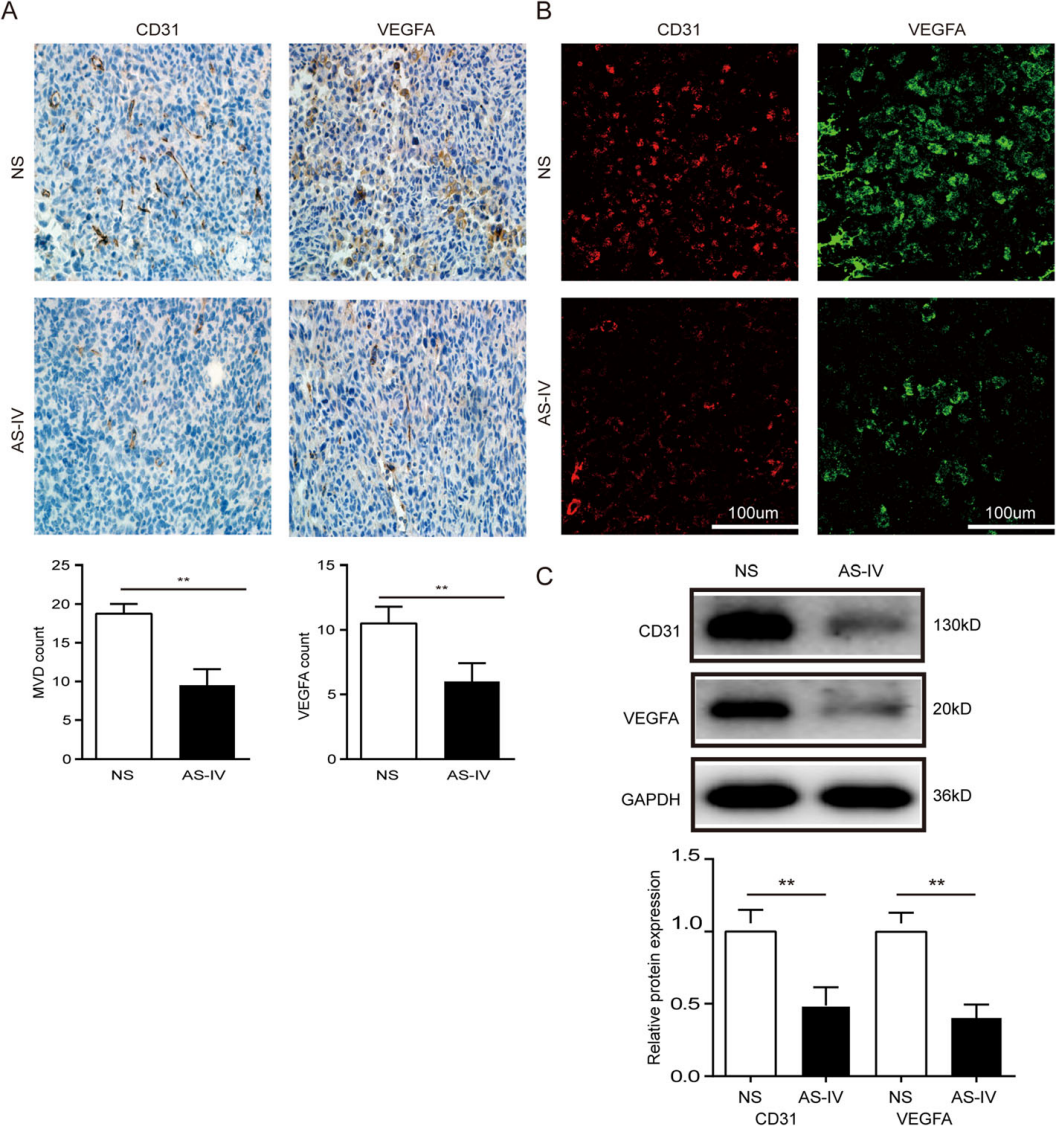
AS-IV inhibited tumor vessel maturation. **a–c** The levels of CD31 and VEGFA were measured by IHC, immunofluorescence, and western blot. Data are presented as the mean ± SEM. *N* = 6–7 animals per group. Compared to the NS group, **p < 0.01

To confirm our in vitro results, we next determined whether AS-IV had an in vivo effect on lung cancer growth in addition to whether it inhibits the occurrence of M2 polarization of macrophages. The subcutaneous mouse model was established by subcutaneous injection of LLC cells. AS-IV inhibited tumor growth and prolonged the survival time in the LLC lung cancer model compared to the NS control group (*p* < 0.01, Fig. 6a–d). In addition, the number of lung metastasis nodules was significantly reduced in the AS-IV treatment group compared to the NS control group (*p* < 0.01, Fig. 6e). Because the status of tumor vessels contributes to tumor metastasis, we also analyzed the impact of AS-IV on vessel density and maturation. CD31 and VEGFA protein expression was confirmed by immunochemistry, immunofluorescence, and western blot. As shown in Fig. 7a–c, micro-vessel density (MVD), as indicated by CD-31 staining, was significantly reduced by AS-IV treatment (*p* < 0.01), and VEGFA expression in tumor tissues was also reduced in the AS-IV treatment group (*p* < 0.01, Fig. 7a–c). Together, these results suggest that AS-IV exerts antitumor effects and suppresses lung cancer metastasis and angiogenesis in vivo.

**Fig. 8:**
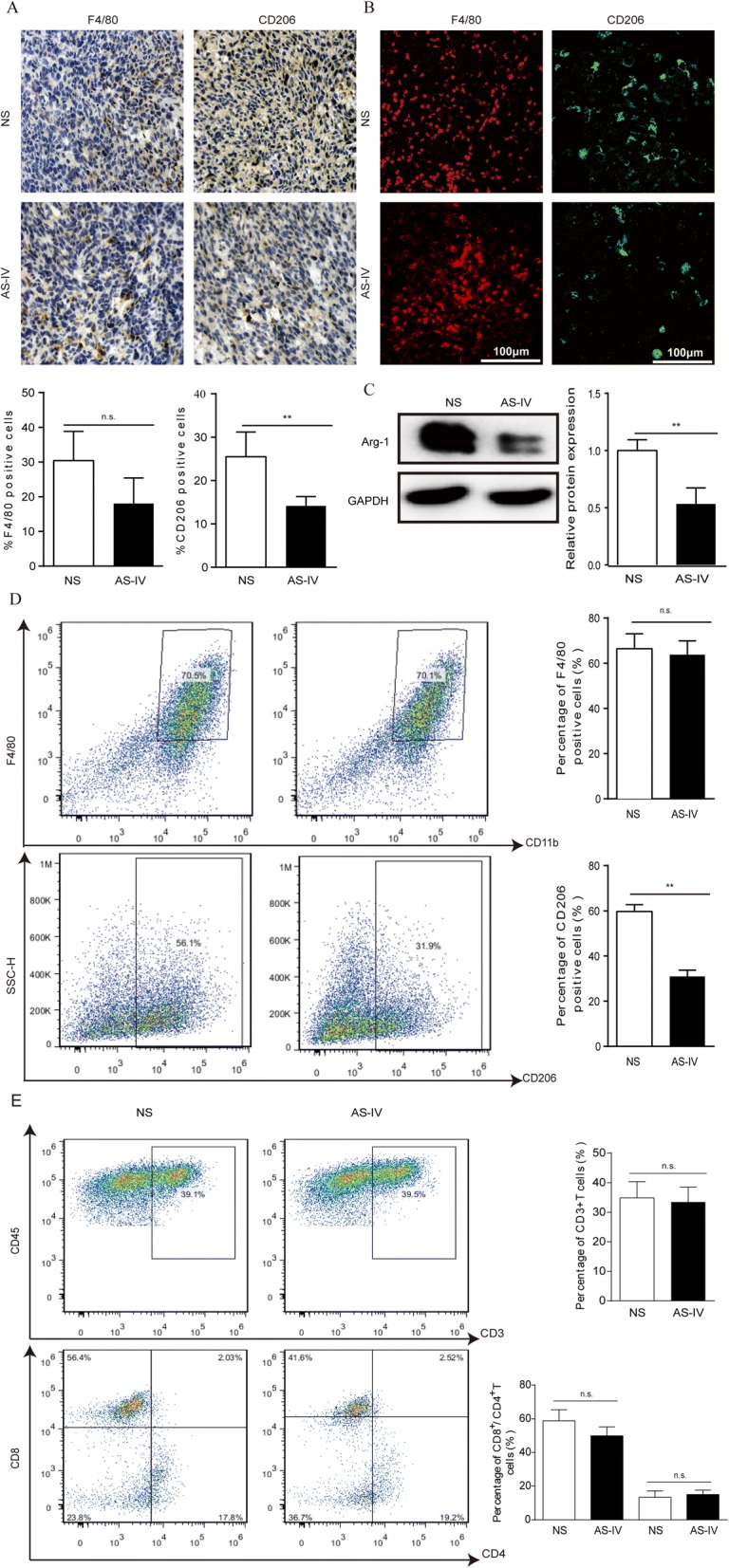
AS-IV suppressed M2 macrophage polarization in the subcutaneous mouse model. (**a** and **b**) The infiltrated macrophages in tumor tissues were stained with the macrophage marker F4/80 and the M2 marker CD206 by IHC and immunofluorescence, and the quantified data are shown. (**c**) Western blot analysis of Arg-1 expression in tumor tissues. (**d**) Macrophages from tumor tissue were stained with antibodies against F4/80 and CD206 and analyzed using flow cytometry. (**e**) T cells from tissues were stained with antibodies against CD3, CD4, and CD8 and assayed by flow cytometry. Data are presented as the mean ± SEM. N = 6–7 animals per group. Compared to the NS group, **p < 0.01, *p < 0.05, n.s., no significant difference

To determine whether the antitumor effect of AS-IV is associated with regulation of macrophage polarization, we analyzed M2 macrophages by immunohistochemistry, confocal microscopy, flow cytometry, and western blot. AS-IV did not induce any obvious change in F4/80-positive TAM accumulation, but it significantly decreased CD206-positive cell infiltration (*p* > 0.05 and *p* < 0.01, respectively; Fig. 8a–d). As observed in the intravenous lung cancer model, AS-IV treatment did not alter the percentage of macrophages but did significantly reduce the number of M2 macrophages (*p* > 0.05 and *p* < 0.01, respectively, Additional file 3: Figure S3). It is worth noting that AS-IV had no effect on T cells in the LLC-bearing mice (*p* > 0.05, Fig. 8e). Together, these results suggest that AS-IV exerts its antitumor effect by inhibiting M2 polarization.

### AS-IV was not toxic to myeloid function or liver or kidney function

To determine whether AS-IV has side effects on myeloid function, hematological parameters from the retro-orbital plexus were measured. As shown in Table 1, treatment with AS-IV had no effects on the hematological parameters, including white blood cells, neutrophils, monocytes, leukocytes, red blood cells, neutrophil-lymphocyte ratio, platelet-lymphocyte ratio, mean corpuscular hemoglobin, mean corpuscular volume, or hemoglobin (*p* > 0.05 for all). These results indicated that AS-IV did not trigger any acute cytotoxic injury.

In addition, to further identify any effect of AS-IV on liver and kidney function, alanine transaminase, glutamic oxaloacetic transaminase, blood urea nitrogen, creatinine, and alkaline phosphatase were measured. As shown in Table 2, no significant differences were observed between treated and untreated mice.

**Table 2:** Effects on liver and kidney function

	NS	AS-IV	*p* value
ALT (U/L)	44.48 ± 3.63	46.78 ± 3.70	0.70
GOT (U/L)	143.90 ± 9.49	145.80 ± 4.93	0.86
BUN (mg/dl)	34.69 ± 0.47	36.38 ± 0.59	0.06
CRE (μmol/L)	95.44 ± 2.48	96.75 ± 1.34	0.78
ALP (U/L)	142.5 ± 16.12	161.2 ± 10.96	0.36

All of the data are presented as the mean ± SEM. N = 5~ 6 animals per group

Abbreviation: *ALT* alanine transaminase, *GOT* glutamic oxaloacetic transaminase, *BUN* blood urea nitrogen, *CRE* creatinine, *ALP* alkaline phosphatase, *SEM* standard error of mean

## Discussion

In the present study, we have shown that AS-IV has inhibitory effects on M2 macrophage polarization. We showed that M2 macrophages enhanced lung cancer growth, migration, and invasion and led to reduced survival in tumor-bearing mice, all of which were alleviated by AS-IV treatment. Also, the increased numbers of M2 macrophages in tumor tissue was attenuated by AS-IV, and genes associated with migration and angiogenesis in macrophages were also significantly inhibited by AS-IV. These results are primarily due to the effect of AS-IV on suppressing M2 polarization.

It is well known that macrophages have divided loyalties and can change their functions in response to the TME. When exposed to Th2 cytokines, IL-4, and/or IL-13, M0 macrophages differentiate into the “alternatively activated” M2 macrophages showed stereotypic alterations in cell surface marker expression [18, 19], and this is in line with our results that macrophages stimulated with IL-4 and IL-13 had increased CD206 expression (Fig. 1). Clinical studies and experimental evidence suggest that M2 macrophages are responsible for tumor-promoting activities, including tumor-associated angiogenesis; tumor initiation, progression, and metastasis; intravasation; and suppression of antitumor immune responses. We obtained similar results showing that M2 macrophages supported the invasion and migration of A549 and H1299 tumor cells as well as vascular remodeling (Fig. 3).

There is growing evidence that depletion of macrophages reduces tumor growth and metastasis [20]. M2 macrophages have been the primary target of cytoreductive therapies, and immunotherapy and therapeutic strategies aiming to reduce the proportion of M2 macrophages or to shift M2 macrophages to M1 macrophages have been suggested to inhibit both tumor growth and metastasis [21, 22]. The present study has further supported this conclusion. In our study, AS-IV exerted remarkable suppression on M2 polarization in vivo and in vitro and clearly affected tumor development. It is now well recognized that inflammation is the causal factor for carcinogenesis, and macrophage polarization presents an entry point into the inflammatory microenvironment [23]. Numerous studies have demonstrated that AS-IV has anti-inflammatory activity and that it protects against inflammatory processes. In a murine model of asthma, AS-IV inhibited the production of proinflammatory cytokines and chemokines by inflammatory cells and ameliorated the immune response [24], and in a rat model of acute ischemic kidney injury AS-IV mediated the inflammatory response by inhibiting NF-κB expression [25]. NF-κB signaling is crucial for the inflammatory phenotype [26], and ablation of NF-κB in myeloid cells attenuates inflammation and inhibits tumor development and progression [27]. Consistent with previous findings [26, 27], our observations revealed that AS-IV clearly ameliorated cancer-associated inflammation, decreased the expression of inflammatory factors such as TGF-β and IL-10 (Fig. 3d), and suppressed M2 macrophage polarization. In addition to our cell line study, our in vivo studies further demonstrated that AS-IV decreased the density of M2 macrophages, as highlighted by the reduced numbers of F4/80-positive cells and CD206-positive cells (Fig. 8). In addition to macrophages, T cells play a crucial role in innate immunity for cancer surveillance; however, our results show that T cells were not affected by AS-IV in the subcutaneous LLC mouse model (Fig. 8i).

There is evidence of a role for reactive oxygen species (ROS) in macrophage differentiation [28]. ROS are generated in the early stage of monocyte-macrophage differentiation, and global ablation of ROS inhibits M2 macrophage polarization [28]. AS-IV has an inhibitory effect on ROS production [29], and this further supports the conclusion that AS-IV has an effect on the differentiation of monocytes into M2 macrophages.

Blood vessels are essential elements for all solid tumor growth, and tumor cells always recruit numerous growth factors and cytokines from the TME to support their growth [30]. TAMs in the TME facilitate angiogenesis by secreting pro-angiogenic factors [31]. In this study, both in vitro experiments and animal tumor models provided evidence that AS-IV decreased the numbers of TAMs based on the reduced levels of angiogenic and tumor growth factors (Fig. 3d and Fig. 7). These results provide new insights into how AS-IV functions as a key factor in inhibiting angiogenesis by abrogating M2 polarization in models of lung cancer.

AMPK is an evolutionarily conserved serine/threonine kinase consisting of three subunits – the regulatory β (β1, β2) and γ (γ1, γ2, γ3) subunits and the catalytic α (α1, α2) subunit – and it is regarded as an energy sensor for the regulation of energy homeostasis and the response to metabolic stress [32, 33]. Recent studies have shown that AMPK also acts in the suppression of inflammatory responses [34, 35], and the phosphorylation of the threonine 172 residue within the catalytic subunit (p-AMPK) plays a pivotal role in its activity. However, the role of AMPK in macrophage differentiation is still controversial. One study found that p-AMPK/AMPK expression was increased in LPS-treated RAW 264.7 cells, while other reports in contrast have suggested that AMPK in macrophages is rapidly activated by anti-inflammatory cytokines such as IL-4 and TGF-β [36, 37]. In our study, p-AMPK levels were significantly elevated after stimulation by IL-4/IL-13 in a time-dependent manner, and after knockdown of AMPK the expression of CD206 was decreased (Fig. 4). In accordance with previous studies [36, 37], these results indicate that AMPK is involved in M2 macrophage polarization. AS-IV induced a dramatic decrease in p-AMPK level, suggesting that the inhibition of AMPK was negatively associated with M2 polarization.

## Conclusions

In summary, we report here that AS-IV reduces the invasion, migration, and angiogenesis of lung cancer by blocking the M2 polarization of macrophages partially through the AMPK signaling pathway. Our findings suggest that TAMs are a potential new target in cancer therapy and provide new insights regarding the therapeutic potential of AS-IV for lung cancer treatment.

## Additional files

Additional file 1: **Figure S1.** Different concentrations of AS-IV were used to inhibit M2 macrophage polarization. (A) THP-1 cells were treated with IL-4/IL-13 and different concentrations of AS-IV for 48 h, and the percentage of M2 macrophages was measured by flow cytometry. (B) To test the toxicity of AS-IV, macrophages were treated with different concentrations of AS-IV for 48 h and then subjected to the MTT assay. Data are presented as the mean ± SEM from three independent experiments. Compared to 0 μM AS-IV, **p < 0.01, *p < 0.05, n.s., no significance. (TIF 845 kb)

Additional file 2: **Figure S2.** DMSO had no effect on M2 polarization. To exclude any influence of DMSO, DMSO in the same volume as the experiments with AS-IV was added to macrophages for 48 h. (A) Representative genes of M1 and M2 macrophages were analyzed by QT-PCR. (B) CD86 (an M1 macrophage marker) and CD206 (an M2 macrophage marker) were evaluated by flow cytometry. Data are presented as the mean ± SEM from three independent experiments. Compared to M0, **p < 0.01, *p < 0.05, n.s., no significance; compared to M2, ^##^*p* < 0.01, ^#^*p* < 0.05, n.s., no significance. (TIF 1678 kb)

Additional file 3: **Figure S3.** AS-IV reduced the numbers of M2 macrophages in the lung metastases. In the intravenous model, the percentages of F4/80-positive and CD206-positive cells were calculated by flow cytometry. Data are presented as the mean ± SEM. *N* = 5 animals per group. Compared to the NS group, **p < 0.01, n.s., no significant difference. (TIF 320 kb)

## Abbreviations

ALP: Alkaline phosphatase
ALT: Alanine transaminase
AMPK: Adenosine monophosphate-activated protein kinase
ANOVA: Way analysis of variance
Arg-1: Arginase-1
AS-IV: Astagaloside IV
BUN: Blood urea nitrogen
COX-2: Cyclooxygenase-2
CRE: Creatinine
DMSO: Dimethyl sulfoxide
ELISA: Enzyme linked immunosorbent assay
GOT: Glutamic oxaloacetic transaminase
HGB: Hemoglobin
HRP: Horseradishperoxidase
IHC: Immunohistochemistry
LLC: Lewis lung cancer
LPS: Lipopolysaccharide
M2-CM: Conditioned medium from M2-like macrophages
MCH: Mean corpuscular hemoglobin
MCV: Mean corpuscular volume
MMP: Matrix metalloproteinase
MVD: Micro-vessel density
NLR: Neutrophil-lymphocyte ratio
NS: Normal Saline
PBS: Phosphate buffered solution
PLR: Platelet-lymphocyte ratio
PMA: Phorbol myristate acetate
RBC: Red blood cell
TAMs: Tumor associated macrophages
TCM: Traditional Chinese medicine
TME: Tumor microenvironment
VEGFA: Vascular endothelial growth factor A
