# Correction: Astragaloside IV inhibits lung cancer progression and metastasis by modulating macrophage polarization through AMPK signaling

**DOI:** 10.1186/s13046-023-02643-y

**Published:** 2023-03-23

**Authors:** Fei Xu, Wen-Qiang Cui, Ying Wei, Jie Cui, Jian Qiu, Ling-Li Hu, Wei-Yi Gong, Jing-Cheng Dong, Bao-Jun Liu

**Affiliations:** 1 Department of Integrative Medicine, Huashan Hospital, Fudan University, Shanghai, China; 2 Institutes of Integrative Medicine, Fudan University, Shanghai, China; 3 Department of Integrative Medicine and Neurobiology, State Key Laboratory of Medical Neurobiology, Institute of Acupuncture Research, School of Basic Medical Science, Fudan University, Shanghai, China

**Correction:** ***BMC Plant Biol*** **37, 207 (2018)**

**https://doi.org/10.1186/s13046-018-0878-0**

Following publication of the original article [1], the author identified an error in the images of Fig. 2 and Fig. 3, specifically:

- Figure 2a - CD86-M0 and M0+AS-IV, CD206-M0 and M0+AS-IV
- Figure 3b - H1299 M0+AS-IV

Furthermore, under Real-time PCR assay (RT-PCR) of Methods section Table 1 citation has to be replaced with Supplementary Table 1. The correct sentence should read as:

‘The sequences of the primers used for the RT-PCR are listed in Supplementary Table 1.’

The correct Fig. 2 and Fig. 3 are given below:

**Fig. 2:**
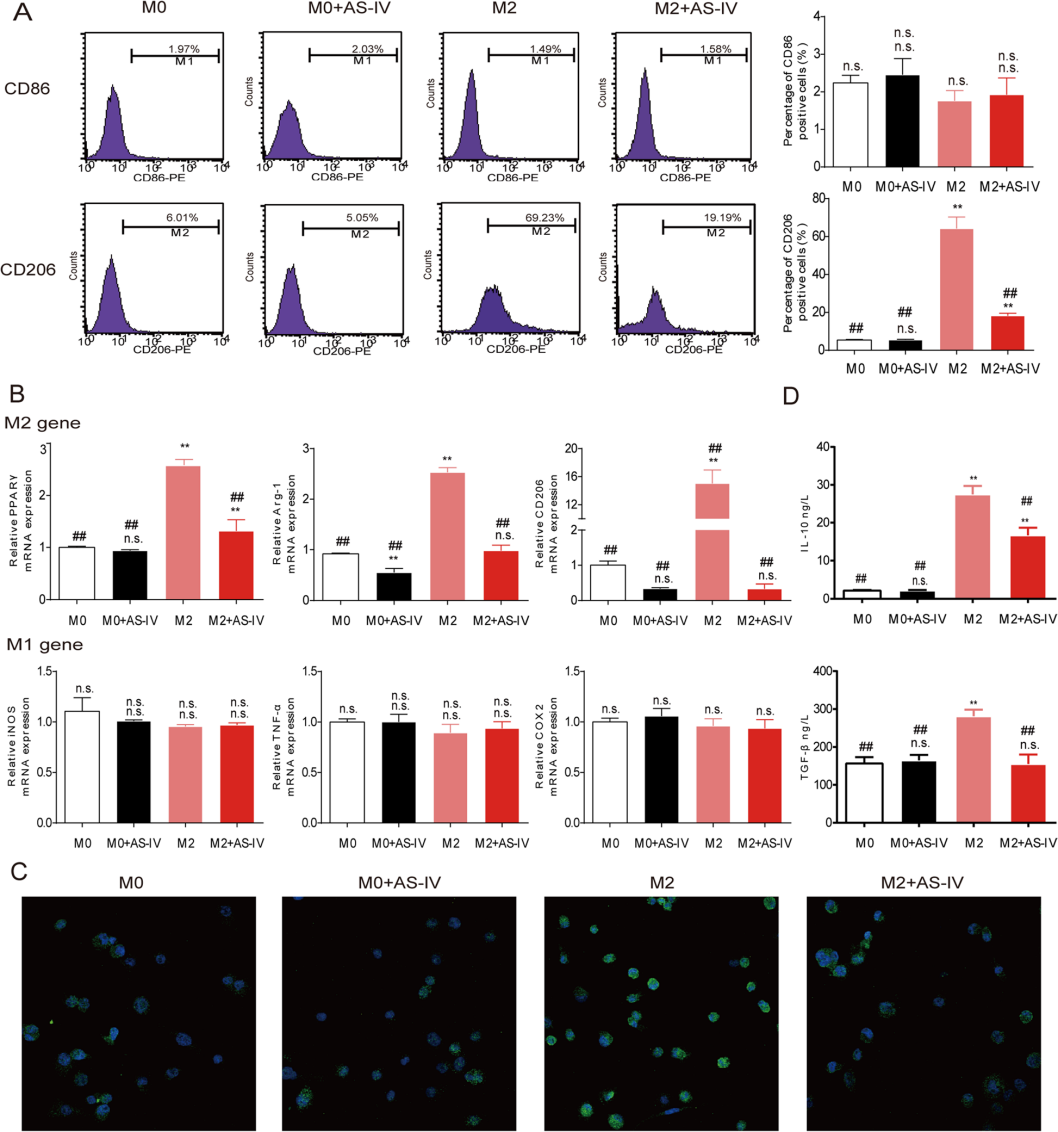
AS-IV inhibited macrophage M2 polarization. Macrophages derived from THP-1 cells were stimulated with IL-4/IL-13 with or without AS-IV (80 μM) for 48 h. **a** Flow cytometry was used to quantify the expression of CD206, an M2 macrophage marker, and CD86, an M1 marker. **b** QT-PCR was performed to detect gene levels in M2 and M1 macrophages. M2-associated genes included *PPARγ*, *Arg-1*, and *CD206*, and M1-associated genes included *iNOS*, *TNF-α*, and *COX2*. **c** Representative images of M0 and M2 macrophages with and without AS-IV stained with antibodies against Arg-1 (green) and with DAPI (blue). **d** The levels of IL-10 and TGF-β in the cell culture supernatants were measured by ELISA. Data are presented as the mean ± SEM from three independent experiments. Compared to M0, **p < 0.01, *p < 0.05, and n.s., no significance; compared to M2, ##*p* < 0.01, #*p* < 0.05, and n.s., no significance

**Fig. 3:**
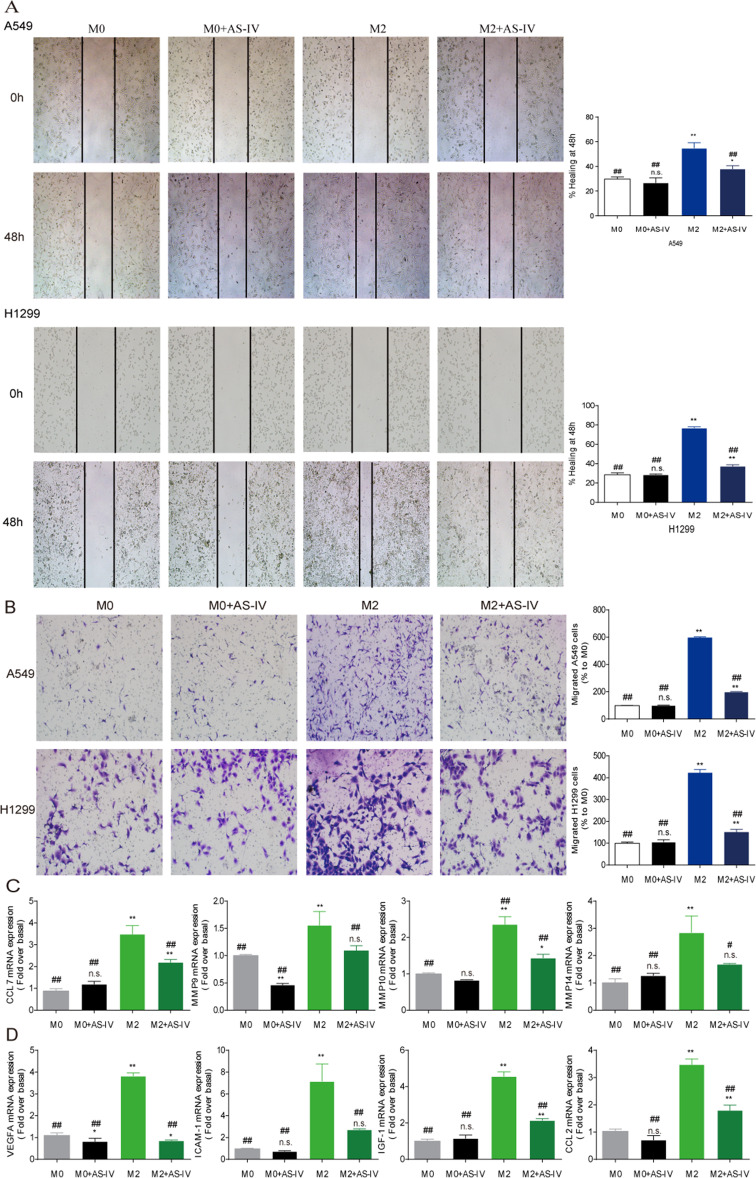
AS-IV inhibited the migration and invasion of tumor cells. Macrophages derived from THP-1 were stimulated with IL-4/IL-13 with or without AS-IV (80 μM) for 48 h, and the conditioned medium (CM) was collected. A549 and H1299 cells were cultured with different CM. **a** and **b** The effect of AS-IV on A549 and H1299 invasion and migration was evaluated by wound scratch assay and trans-well assay, respectively. **c** and **d** The mRNA levels of genes associated with migration and angiogenesis in macrophages. Data are presented as the mean ± SEM from three independent experiments. Compared to M0, **p < 0.01, *p < 0.05, and n.s., no significance; compared to M2, ^##^*p* < 0.01, ^#^*p* < 0.05, and n.s., no significance

## Supplementary Information

**Additional file 1: Supplementary Table 1.** Sequences of the primers used for PCR.
