# Supplementary figures and images for: Astragaloside IV inhibits lung cancer progression and metastasis by modulating macrophage polarization through AMPK signaling
Source: J Exp Clin Cancer Res. 2018 Aug 29;37:207. doi: 10.1186/s13046-018-0878-0 (PMC6116548; doi:10.1186/s13046-018-0878-0)

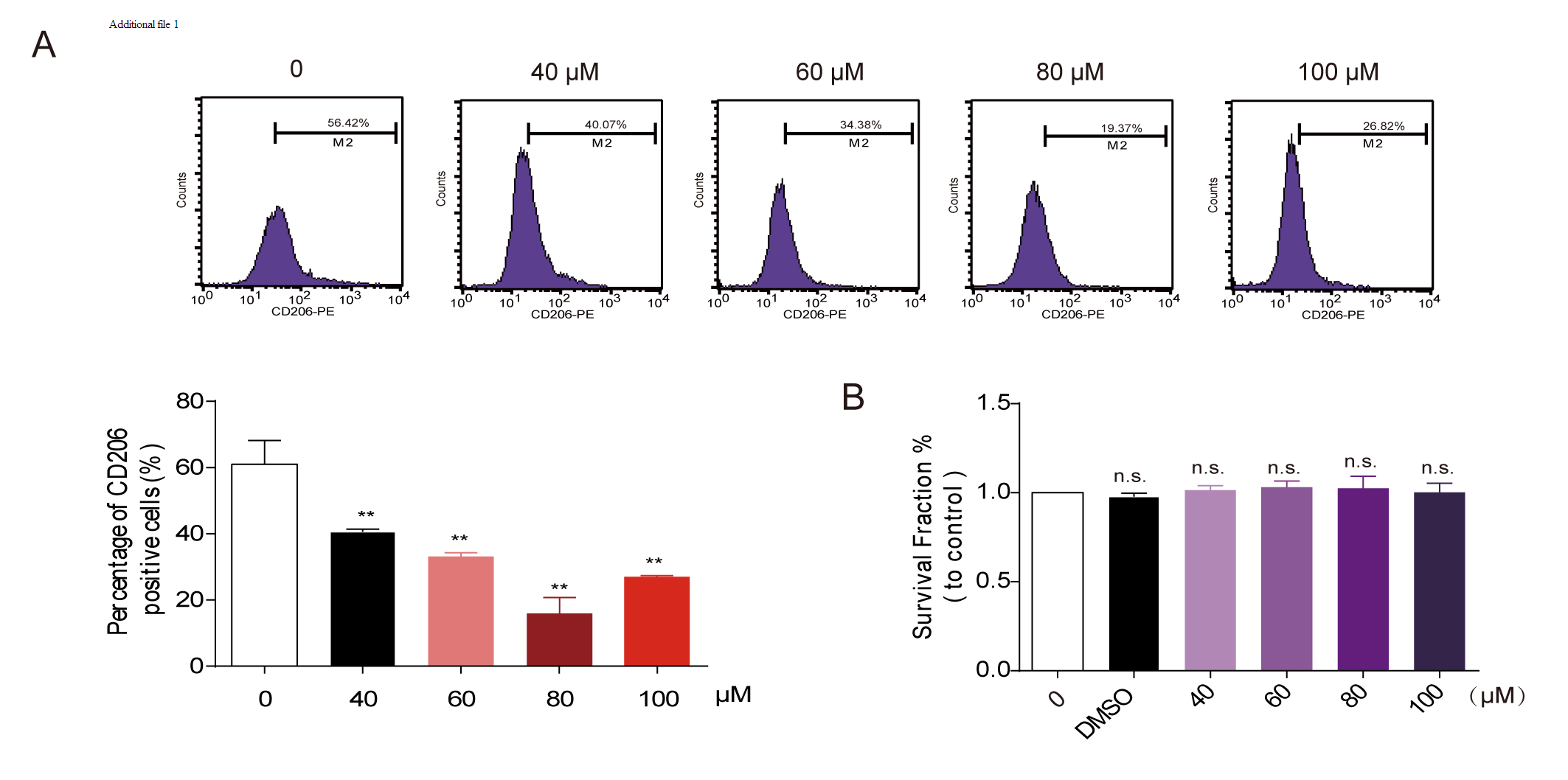

Supplement: Supplementary file 1 — Figure S1. Different concentrations of AS-IV were used to inhibit M2 macrophage polarization. (A) THP-1 cells were treated with IL-4/IL-13 and different concentrations of AS-IV for 48 h, and the percentage of M2 macrophages was measured by flow cytometry. (B) To test the toxicity of AS-IV, macrophages were treated with different concentrations of AS-IV for 48 h and then subjected to the MTT assay. Data are presented as the mean ± SEM from three independent experiments. Compared to 0 μM AS-IV, **p < 0.01, *p < 0.05, n.s., no significance. (TIF 845 kb) [file 13046_2018_878_MOESM1_ESM.tif]

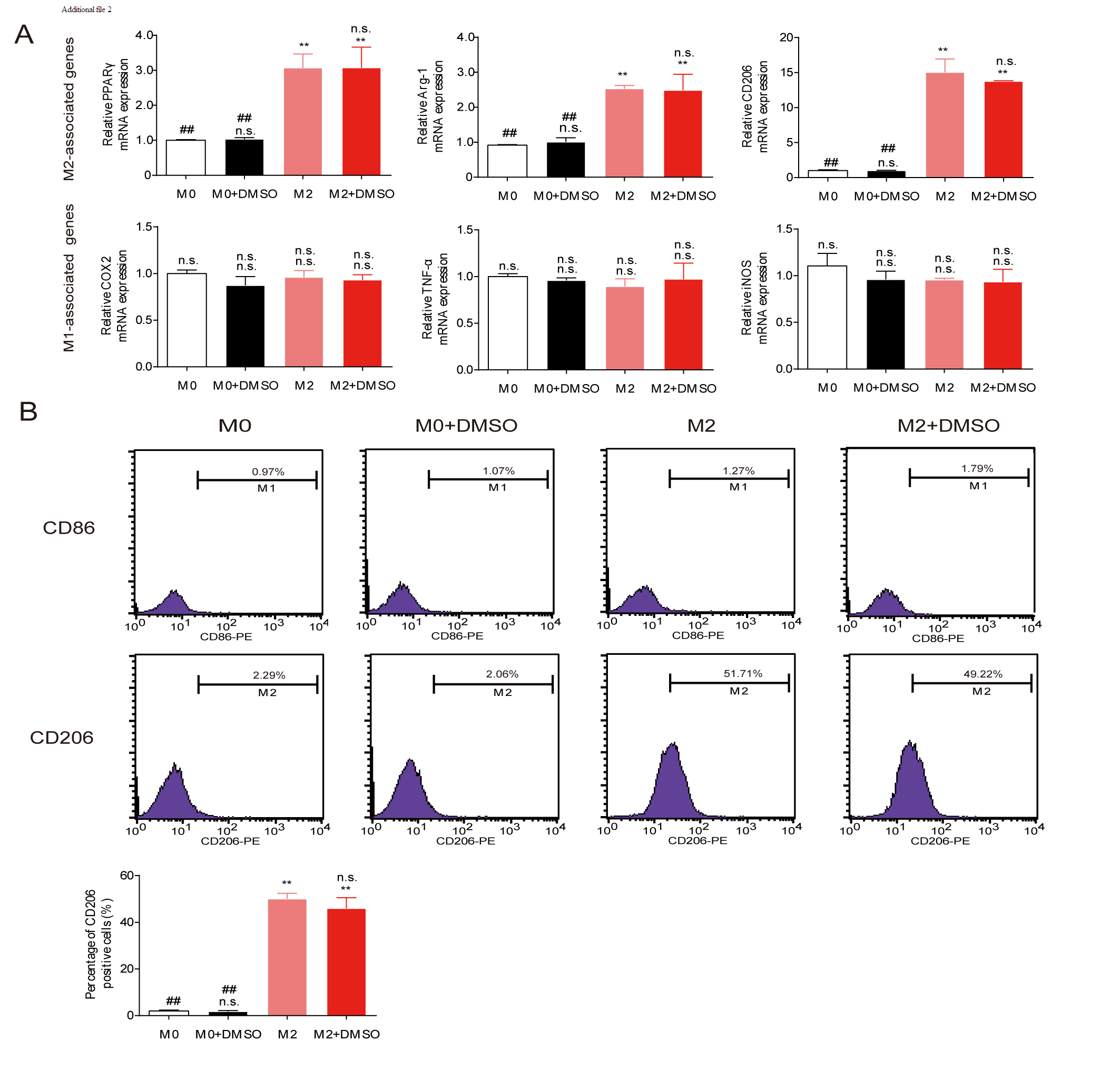

Supplement: Supplementary file 2 — Figure S2. DMSO had no effect on M2 polarization. To exclude any influence of DMSO, DMSO in the same volume as the experiments with AS-IV was added to macrophages for 48 h. (A) Representative genes of M1 and M2 macrophages were analyzed by QT-PCR. (B) CD86 (an M1 macrophage marker) and CD206 (an M2 macrophage marker) were evaluated by flow cytometry. Data are presented as the mean ± SEM from three independent experiments. Compared to M0, **p < 0.01, *p < 0.05, n.s., no significance; compared to M2, ##p < 0.01, #p < 0.05, n.s., no significance. (TIF 1678 kb) [file 13046_2018_878_MOESM2_ESM.tif]

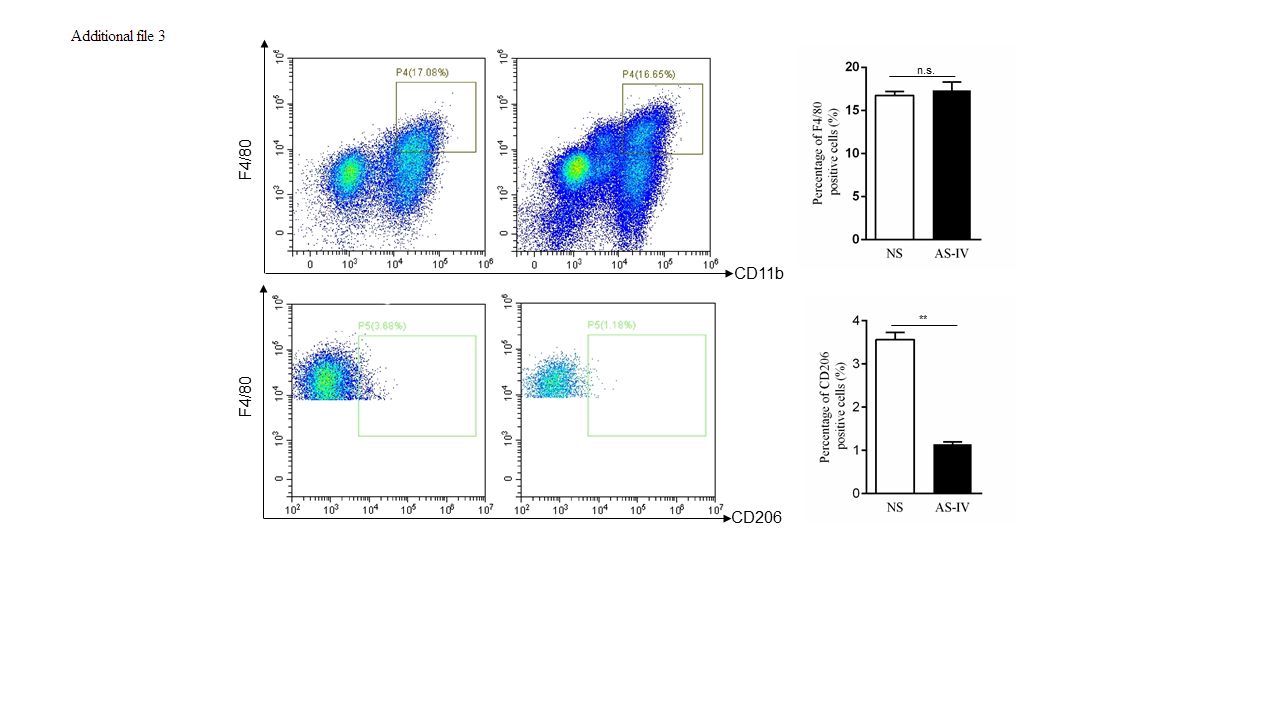

Supplement: Supplementary file 3 — Figure S3. AS-IV reduced the numbers of M2 macrophages in the lung metastases. In the intravenous model, the percentages of F4/80-positive and CD206-positive cells were calculated by flow cytometry. Data are presented as the mean ± SEM. N = 5 animals per group. Compared to the NS group, **p < 0.01, n.s., no significant difference. (TIF 320 kb) [file 13046_2018_878_MOESM3_ESM.tif]
