# Supplementary material for: Correction: Astragaloside IV inhibits lung cancer progression and metastasis by modulating macrophage polarization through AMPK signaling
Source: J Exp Clin Cancer Res. 2023 Mar 23;42:70. doi: 10.1186/s13046-023-02643-y (PMC10035129; doi:10.1186/s13046-023-02643-y)
Supplement: Supplementary file 1 — Additional file 1: Supplementary Table 1. Sequences of the primers used for PCR. [file 13046_2023_2643_MOESM1_ESM.docx]

**Supplementary Table1. Sequences of the primers used for PCR**

| Gene Name | 5’ –3’ (Forward) | 5’ –3’ (Reverse) |
| --- | --- | --- |
| CD206 | GGGTTGCTATCACTCTCTATGC | TTTCTTGTCTGTTGCCGTAGTT |
| Arg-1 | CAACCCGAGAAACACATCCT | ATATACACACTGCGCCCACA |
| COX2 | TGCTGTGGAGCTGTATCCTG | CGGGAAGAACTTGCATTGAT |
| TNF-α | TCCTTCAGACACCCTCAACC | CACATTCCTGAATCCCAGGT |
| iNOS | CTGAGGAGGAAAGCAAGGTG | GCCCAAGCAGAAACTGAGAC |
| IL-10 | GCACAGCTCCAAGAGAAAGG | ATAGAGTCGCCACCCTGATG |
| CCL17 | ACGAGGTCAGGAGATCGAGA | CAGCCTCCCAAGTAGCTGAG |
| CCL18 | TGTAAGTGCCAGTGCTCCTG | TCCCCTGGAGTCCTTTACCT |
| CCL22 | CTGAACCCAGCCTGACAAAT | CTGGATGACACTGAGCTGGA |
| TGF-β | TCTCGAACTCCTGACCTCGT | TGCACAAGCTGGTCTTGAAC |
| MMP-9 | CTCGAACTTTGACAGCGACA | TTCACGTCGTCCTTATGCAA |
| IL-1β | AACCCAAGGAAGGGCTCTAA | CAGCCTGTGTCTCACTGGAA |
| CCR7 | CCAGAATCCCTGGCTTTACA | TAGCCCTCTTCCCGTATCCT |
| AMPKα | CAACTATCGATCTTGCCAAAGG | AACAGGAGAAGAGTCAAGTGAG |
| ICAM-1 | TGCAAGAAGATAGCCAACCAAT | GTACACGGTGAGGAAGGTTTTA |
| IGF-1 | TTAGGAGGCTGAGGCAGGAG | GACCTGGCACATGGTAAGCA |
| MMP-14 | CCTACAGCCGCTTCCTGAAC | ATTAGCTGCACGTGGTGGTG |
| MMP-10 | TGTGGAGTTCCTGACGTTGG | GCCTGGAGAATGTGAGTGGA |
| VEGFA | GTGCCCATTCTCCTGACATT | AGTCTTGCCTCCCTGACTGA |
| CCL2 | TCTGTGCCTGCTGCTCATAG | CAGAGCTGGAATCCTGGAAG |
| GAPDH | AAATGGTGAAGGTCGGTGTG | AGGTCAATGAAGGGGTCGTT |
| PPARγ | TACTGTCGGTTTCAGAAATGCC | GTCAGCGGACTCTGGATTCAG |
